# Dynamics of T2* and deformation in the placenta and myometrium during pre-labour contractions

**DOI:** 10.1038/s41598-022-22008-3

**Published:** 2022-11-03

**Authors:** Jana Hutter, Vikram Kohli, Neele Dellschaft, Alena Uus, Lisa Story, Johannes K. Steinweg, Penny Gowland, Joseph V. Hajnal, Mary A. Rutherford

**Affiliations:** 1 Centre for the Developing Brain, King’s College London, London, UK; 2 Department of Biomedical Engineering, School of Biomedical Engineering & Imaging Science, King’s College London, London, UK; 3 GKT School of Medicine, King’s College London, London, UK; 4 Sir Peter Mansfield Imaging Centre, University of Nottingham, Nottingham, UK; 5 Academic Women’s Health Department, King’s College London, London, UK; 6 Fetal Medicine Department, GSTT, London, UK; 7 Department of Cardiovascular Imaging, School of Biomedical Engineering & Imaging Science, King’s College London, London, UK

**Keywords:** Magnetic resonance imaging, Paediatric research

## Abstract

Pre-labour uterine contractions, occurring throughout pregnancy, are an important phenomenon involving the placenta in addition to the myometrium. They alter the uterine environment and thus potentially the blood supply to the fetus and may thus provide crucial insights into the processes of labour. Assessment in-vivo is however restricted due to their unpredictability and the inaccessible nature of the utero-placental compartment. While clinical cardiotocography (CTG) only allows global, pressure-based assessment, functional magnetic resonance imaging (MRI) provides an opportunity to study contractile activity and its effects on the placenta and the fetus in-vivo. This study aims to provide both descriptive and quantitative structural and functional MR assessments of pre-labour contractions in the human uterus. A total of 226 MRI scans (18–41 weeks gestation) from ongoing research studies were analysed, focusing on free-breathing dynamic quantitative whole uterus dynamic T2* maps. These provide an indirect measure of tissue properties such as oxygenation. 22 contractile events were noted visually and both descriptive and quantitative analysis of the myometrial and placental changes including volumetric and T2* variations were undertaken. Processing and analysis was successfully performed, qualitative analysis shows distinct and highly dynamic contraction related characteristics including; alterations in the thickness of the low T2* in the placental bed and other myometrial areas, high intensity vessel-like structures in the myometrium, low-intensity vessel structures within the placental parenchyma and close to the chorionic plate. Quantitative evaluation shows a significant negative correlation between T2* in both contractile and not-contractile regions with gestational age (*p* < 0.05) as well as a significant reduction in T2* during contractions. The T2* values in the myometrium were however not correlated to gestational age (*p* > 0.5). The quantitative and qualitative description of uterine pre-labour contractions including dynamic changes and key characteristics aims to contribute to the sparsely available in-vivo information and to provide an in-vivo tool to study this important phenomenon. Further work is required to analyse the origins of these subclinical contractions, their effects in high-risk pregnancies and their ability to determine the likelihood of a successful labour. Assessing T2* distribution as a marker for placental oxygenation could thus potentially complement clinically used cardiotocography measurements in the future.

## Introduction

**Figure 1:**
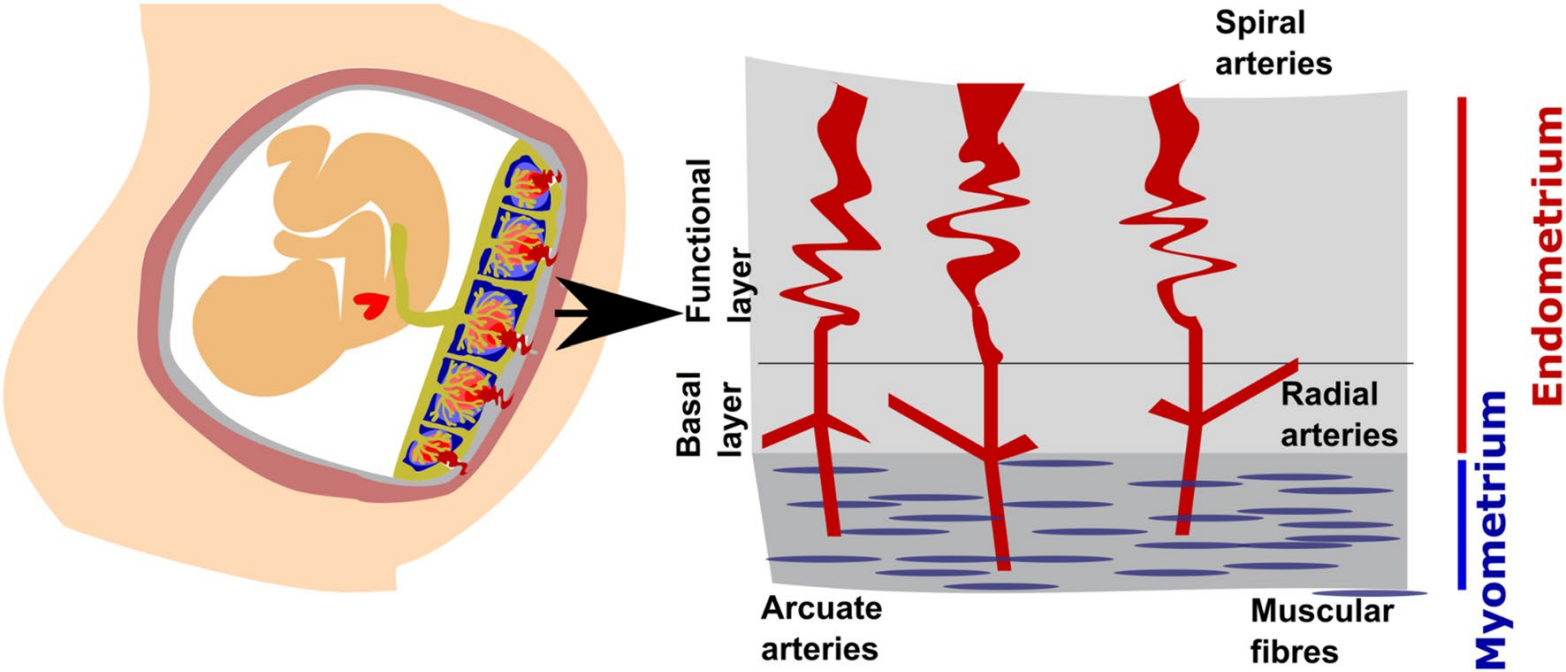
Schemata of the uterus and placental bed with a focus on the endometrium and myometrium.

Pregnancy is without doubt one of the most fascinating and complex periods in life. A complex cascade of interacting processes are involved in early human development and in the successful birth of a newborn. One important phenomenon are contractions of the uterine muscles, crucial during parturition but starting as early as 6 weeks of gestation, although usually not felt until the late second or third trimester. The human uterus is composed of three layers, the serosa, myometrium and endometrium. The myometrium is a thick, muscular layer of the uterine wall, which contains the arcuate arteries. It is lined by the endometrium, subdivided into a functional layer, responsive to reproductive hormones, and the basal endometrium. During pregnancy, the placenta is attached to the uterine wall and receives its blood supply from the spiral endometrial arteries, originating from the uterine, arcuate and then radial arteries, as illustrated in Fig. 1. The endometrium contains the radial and spiral aspects of the vasculature, with final connections into the decidua and intervillous space. The placenta plays a multitude of crucial roles for the developing fetus, including the delivery of oxygen and nutrients, excretion of fetal waste products, synthesis of hormones and growth factors and acts as a maternal–fetal immune barrier.

The myometrium underneath the placenta, forming, together with the endometrium, part of the placental bed, undergoes a range of dramatic alterations to adapt to its evolving functions during pregnancy—these alterations start following the trophoblastic invasion of the spiral arteries in early gestation to ensure constant low pressure supply to the growing fetus and later prepare for the expulsion of the fetus during labour with contractile muscular activity. Irregular, low intensity, low frequency and often not perceived uterine contractions occur spontaneously throughout pregnancy, referred to as Braxton Hicks contractions and often described as ‘practice contractions’, thought to strengthen the uterine musculature^1^. Their precise aetiology remains unclear and there has been limited scientific literature investigating their effects to date^2^. Importantly, there is a paucity of data regarding the effect of these subclinical contractions on placental function.

Most research utilises pulsed wave doppler flow imaging to study uteroplacental perfusion. Oosterof et al*.* identified marked elevations in the pulsatility index of the uterine arteries during Braxton Hicks contractions, identified with Doppler velocimetry, indicating significant increases in vascular resistance during these uterine contractions^3^. Moreover, research has identified changes in fetal heart-rate patterns during these non-labour contractions in growth-restricted fetuses, further reinforcing the need to gain a deeper understanding of the physiological implications of these contractions in healthy and high risk pregnancies^4^. Various animal models have furthermore been used to study uterine contractions (Malik et al. 2021). While Ultrasound remains the most commonly used imaging modality for in-utero research, in recent years an increase in studies employing Magnetic Resonance Imaging (MRI) has been observed, in order to gain greater insight into the placenta in-vivo. MRI enables not only anatomical studies, but also facilitates the study of specific functions such as perfusion^5^, oxygenation, indirectly via T2* relaxometry^6^ and assessment of cellular density^7^ safely and non-invasively during pregnancy^8^.

Data acquired at multiple echo times allows the calculation of T2* maps which in turn indirectly inform on oxygenation via the Blood Oxygen Level Dependent (BOLD) effect, utilising the paramagnetic properties and thus decreased T2* time of deoxyhaemoglobin^9^. For the placenta, promising results show an inverse relation between mean T2* and gestational age as well as reduced T2* associated with low birth weight^10^, pre-eclampsia^11^ and fetal growth restriction^12^. However, the T2* value does not depend solely on the concentration of deoxyhaemoglobin, but on additional factors including water content, blood volume and surface area, thus enabling only an indirect quantification of oxygenation. In addition, BOLD imaging during maternal hyperoxygenation, to study the dynamics of oxygen uptake, has been previously acquired at a single echo time, thus without absolute quantification but rather assessing relative changes, prior to and during/after hyperoxygenation^13,14^**.**

Recently, contractions have been assessed by a hree studies from Turk et al.^15^, Sinding at al18 and Dellschaft et al. (Dellschaft et al. 2020) using single-echo time BOLD data at 3 T. Turk et al. analysed the influence of both maternal position and contractions on placental parameters . Placental volume and expansion were assessed and showed placental contractions in over 50% of the 24 participants. Just under half of these showed regional volume variation and just over half total placental volume changes.

Sinding et al. reported subclinical contractions in 8 out of 56 healthy participants^16^.Manual ROIs were used to demonstrate reduction in placental volume and a decrease in BOLD signal. Both changes were observed with a time delay of about 30 s.

Most recently, Dellschaft et al. analysed changes in the myometrium of the placental bed as well as in the total recorded placental and uterine volume^17,18^. Whole uterus contractions with no alterations in placental volume were considered to be consistent with Braxton Hicks contractions. Contractions of the placenta and underlying uterine wall contracting independently of the rest of the uterus as “placental pump” contractions.

This study aims to use dynamically resolved functional MRI techniques to assess the impact of subclinical contractions on placental function. We hypothesise that the contraction of the muscles in the myometrium contributes to narrowing of the arteries supplying the placenta thus leading to a dynamically changing supply of highly oxygenated maternal blood to the placenta.

## Materials and Method

### Data acquisition

This project analysed datasets collected prospectively as part of the Placental Imaging Project as approved by the Fulham Research Ethics Committee (REC16/LO/1573) between 2016 and 2022. All experiments were performed in accordance with relevant guidelines and regulations. Informed written consent was obtained from all participants.

Inclusion criteria included women in their 2nd or 3rd trimester and with a singleton pregnancy. Women with contraindications to MRI, such as implants or claustrophobia, were excluded. Individual demographics were recorded including age, body mass index (BMI), medical history, obstetric and antenatal history. Images for these studies were obtained using a clinical 1.5 Tesla Philips Ingenia scanner and a 3 Tesla Philips Achieva scanner (Best, The Netherlands) using a 28 or 32-channel coil respectively.

A T2-weighted Turbo Spin echo sequence in 5–8 orientations covering the uterus was performed. A free breathing multi-echo gradient-echo (MEGE) echo planar imaging pulse sequence was performed on each subject covering the entire uterus in coronal orientation to the mother, with isotropic resolution, and four echo times between 11 and 200 ms. The repetition time was between 4–14 s and between two and a 100 dynamics were acquired. At least one complete set of volumes was acquired per woman. In some cases scans were repeated in women where clear contractile activity was visually identified during the scan. The protocol at 3 T had a resolution of 2.5 mm isotropic, TEs = [13.8, 70.4, 127, 183.6],TR = 12 s and on 1.5 T a resolution of 3 mm isotropic, TE = [11.7,57.3,103.2,149.2], TR = 14 s.

All women were imaged in the supine position with dedicated padding for their lower backs and knees and with ear protection. Temperature measurements were taken both pre and post scanning and the maternal heart rate was continuously monitored throughout the examination. Alongside this, all patients underwent serial blood pressure monitoring as well as pulse oximetry for the entire duration of the scan. Scan times were restricted to 60 min and split into two halves of 30 min each. Delivery parameters were collected where available, such as gestational age at delivery, birthweight centile, sex, mode of delivery, head circumference as well as neonatal and maternal outcomes.

For this study, only datasets with a completed multi-dynamic MEGE sequence obtained on the 1.5 T and 3 T scanner were considered. MEGE data was assessed for contractile activity.

### Processing and Data analysis

The multi-echo gradient echo (MEGE) data was fitted to a mono-exponential decay function as previously used in in-house scripts (Hutter et al. 2019) to obtain T2* maps. The placenta was identified manually on each individual slice of the first dynamic. For these placental-only masks, the maternal uterine wall tissue, or voxels belonging to the placental bed, was excluded as well as the amniotic fluid, blood vessels and fetal structures. For scans that demonstrated contractile activity on the MEGE scans, further segmentation was conducted in both the dynamics with maximal contraction (as defined by the maximal visual changes) and a contraction-free volume wherever available. These segmentations were performed on the contractile area in the placenta as well as the underlying myometrium avoiding any visible vasculature. Segmenting the myometrium in dynamics without contractile activity was not possible due to the thin myometrium in this state.

An extension of rigid SVR reconstruction method with hierarchical deformable registration scheme and structure-based outlier rejection–called DSVR^19^—was performed, allowing for correction of non-rigid deformation. Therefore, a template consisting of the average of 10 volumes taken from the mid-dynamics was used for initial registration. The provided masks were propagated to all dynamics to calculate the Jacobian deformation and the original placental volume. Mean T2* volume as well as histogram skewness and kurtosis were calculated for the entire placental parenchyma based on the histograms of these distributions. Skewness thereby describes the direction (sign) and amount of the histogram tending towards either higher values or lower values and kurtosis the height and peakness of the central peak with a high sharp peak displaying high kurtosis and a flat distribution low kurtosis. These were calculated in Python using the library SciPy.

#### Results

**Figure 2:**
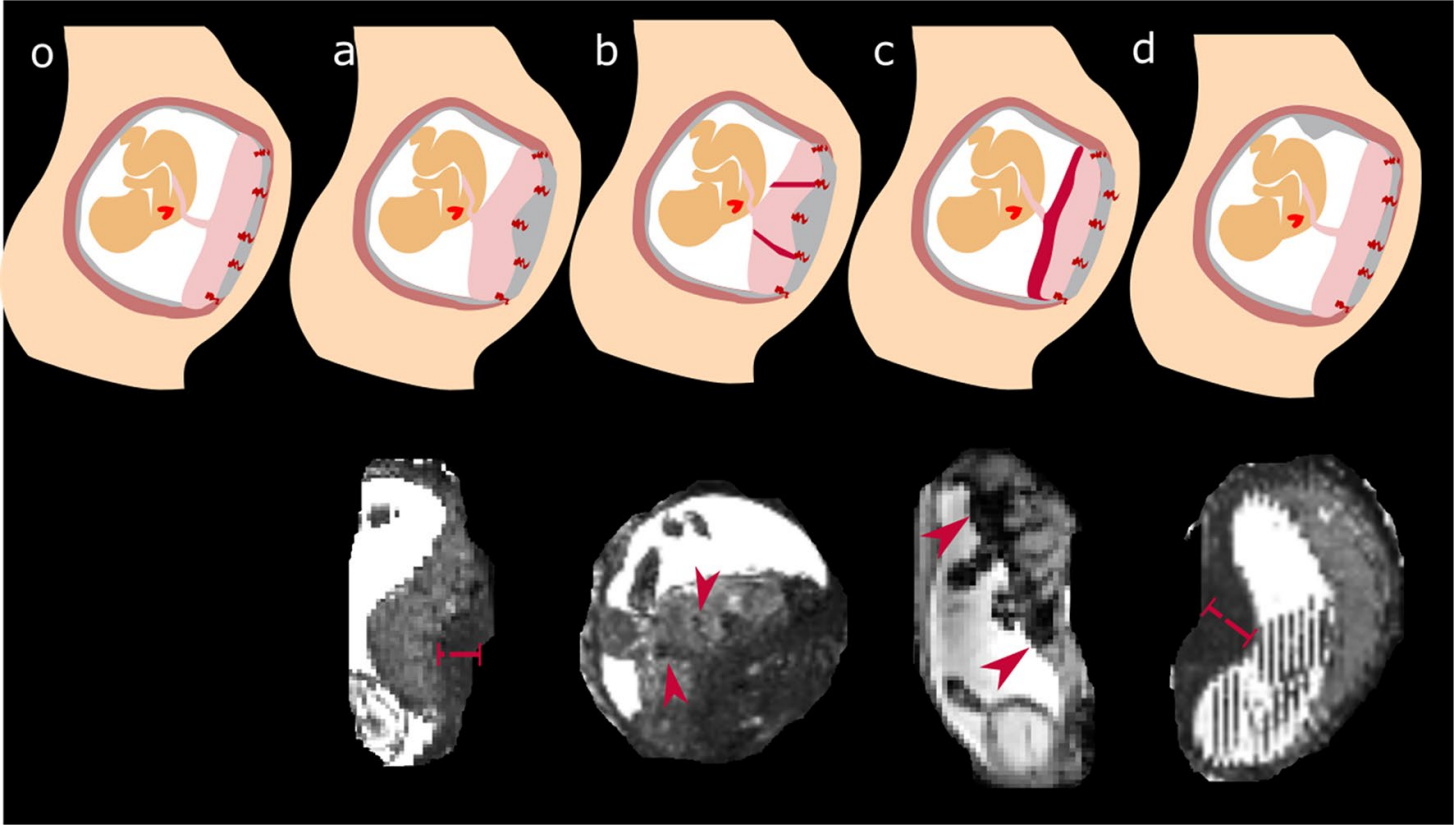
Identified significant changes associated with contractile activity in the uterus are shown in schemata and in coronal or reformatted sagittal views of the MEGE scans. A schemata without signs of contractions (left) is followed by four different identified signs on T2/T2* weighted images (**a**) thickening of the uterine wall underneath the placenta, (**b**) hypointense (on T2) lines in the placental parenchyma, (**c**) hypointense (on T2) rim next to the chorionic plate and (**d**) thickening of the uterine wall in other areas of the uterine wall, not underlying the placenta.The red bars and arrows indicate the changes, in (**a**) the thickening underneath the placenta, in (**b**) the vessel like hypointense bands in the placenta, in (**c**) the hypointense band on the chorionic surface and in (**d**) the thickening of the myometrium distant to the placenta.

The assessment with above mentioned deformation and Jacobians did not robustly identify all visually observed contractions, reflecting their different characteristics as detailed below. The definition of a contraction was thus based on visual impression assessed using the characteristics in Fig. 2, results for the automatic deformation analysis are given below. MEGE datasets were successfully analysed and processed in all 248 scans as outlined above. Contractions were visually identified in the MEGE scans of 22 participants, three of these were scanned at 3 T and 19 at 1.5 T . The cohort statistics for those with identified contractions on the MEGE scan and without identified contractions are given in Table 1., showing that the GA at scan, maternal age, GA and birth weight centile at delivery are all comparable between groups. However, the maternal BMI at the time of booking was significantly higher in the contraction cohort (29 in the contraction cohort versus 24.8 in the no contraction cohort). The results from visual inspection on the non-processed data is presented initially and summarised in Table 2 and then, the data after registration and correction for motion and volume changes is given, allowing in turn the quantification of the dynamics of T2* changes over time in better detail. Finally, quantitative results over GA are given and discussed.

**Table 1:** Cohort statistics of the observed contractions with sufficient temporal T2* data.

	Contractions identified (N = 22) Mean *± *std [min,max]	No contractions identified (N = 226) Mean *± *std [min,max]
Gestational Age @ scan [weeks]	27.86 *± *5.86 [ 18.58 , 37.43 ]	29.01 *± *9.62 [ 31.57 , 37.86 ]
Age [years]	33.27 *± *5.75 [ 18.49 , 43.27 ]	34.45 *± *4.81 [ 18.82 , 47.17 ]
BMI	29.02 *± *5.64 [ 22.04 , 41.81 ]	24.78 *± *3.87 [ 16.80 , 39.47 ]
Gestational Age @ delivery [weeks]	38.72 *± *1.89 [ 33.71 , 41.71 ]	37.45 *± *4.01 [ 24.43 , 41.86 ]
Birth weight [centile]	37.66 *± *24.10 [ 7.93 , 71.93 ]	38.66 *± *23.83 [ 2.30 , 71.76 ]
Diabetes (GDM, DT1, DT2)	N = 4/22 = 18%	N = 10/226 = 4.4%

**Table 2:** Overview over the considered cases of contractile activity and observations on the T2* maps.

ID	Heterogeneity	Chorionic side	Myometrium (placental bed)	Myometrium (not placenta)	Change in shape	Partial/Complete	Migrating	Protocol
1	Homogeneous	hypointense	thin	thick areas	y	C	n	1.5
2	Heterogeneous	hypointense	thick	thick areas	y	P	y	1.5
3	Heterogeneous	hypointense	thick	not covered	y	P	y	1.5
4	Homogeneous	hypointense	thick	not covered	y	P	y	1.5
5	Homogeneous	hyperintense	thick	not covered	y	P	n	1.5
6	Heterogeneous	hypointense	thin	thick	y	C	y	3
7	Heterogeneous	hypointense	thick	thin	y	C	n	1.5
8	Homogeneous	hyperintense	thick	not covered	y	P	y	1.5
9	Heterogeneous	hypointense	thin	not covered	y	P	n	1.5
10	Heterogeneous	hypointense (edge)	thick (edge)	not covered	y	P	n	1.5
11	Heterogeneous	hypointense	thin	not covered	y	P	n	1.5
12	Heterogeneous	hypointense	thin	thick (edge)	y	P	n	1.5
13	Heterogeneous	hypointense (white rim)	thick	not covered	y	P	n	1.5
14	Heterogeneous	hypointense	thick	thin not covered	y	P	n	1.5
15	Heterogeneous	hypointense	thin	thick	y	P	y	3
16	Heterogeneous	hypointense (edge)	thick (edge)	thin	y	P	n	1.5
17	Heterogeneous	hyperintense	thick	thin	y	P	n	1.5
18	Heterogeneous	hypointense	thick	not covered	y	P	n	1.5
19	Heterogeneous	hypointense	thick (edge)	not covered	y	p	n	1.5
20	Heterogeneous	hypointense	thin	not covered	y	p	n	3
21	Heterogeneous	hypointense	thick (edge)	thin	y	p	n	1.5
22	Heterogeneous	hypointense	thick (edge)	thin	y	p	n	1.5

Heterogeneity refers to the overall signal intensity within the placenta, where “heterogeneous” refers to a visible clear observable change such as marked with a blue arrow in Fig. 2 b and c. and homogenous showed no focal placental signal intensity changes. Chorionic side assesses the observable change on the chorionic side of the placenta, most notably whether a dark rim was observed (“hypointense”) (Fig. 2c). The myometrial thickness is observed both underneath the placenta and at a distance. Any changes in shape are recorded. ‘Partial’ or ‘complete’ describes whether the changes are observed in the entire placenta or only in a part of it. Migrating captures whether the thickening observed changed location during the dynamic scan. In the final column, the scanner field strength and protocol is indicated, with 1.5 and 3 representing in turns the 1.5 T and 3 T scanner.

### Different contractile states based on location and pattern of observed changes

The visual inspection of the dynamics revealed changes suggestive of contractions in the placental tissue and the uterine wall in 22 T2*-mapping scans with multiple temporal dynamics. These varied widely in appearance and are here classified by the absence or presence of four criteria defined by both the visibility of increases in thickness within the uterine wall and changes to the T2* signal within the placenta. These are depicted as schemata with example cases in Fig. 2. Criteria (a) a thickening of the myometrium underneath the placenta, (b) increased heterogeneity appearing as dark lines starting from the basal plate, (c) an increase in heterogeneity marked by a decrease in signal close to the chorionic plate and (d) a thickening of the myometrium at a location of the uterine wall distanced from the placenta.

**Figure 3:**
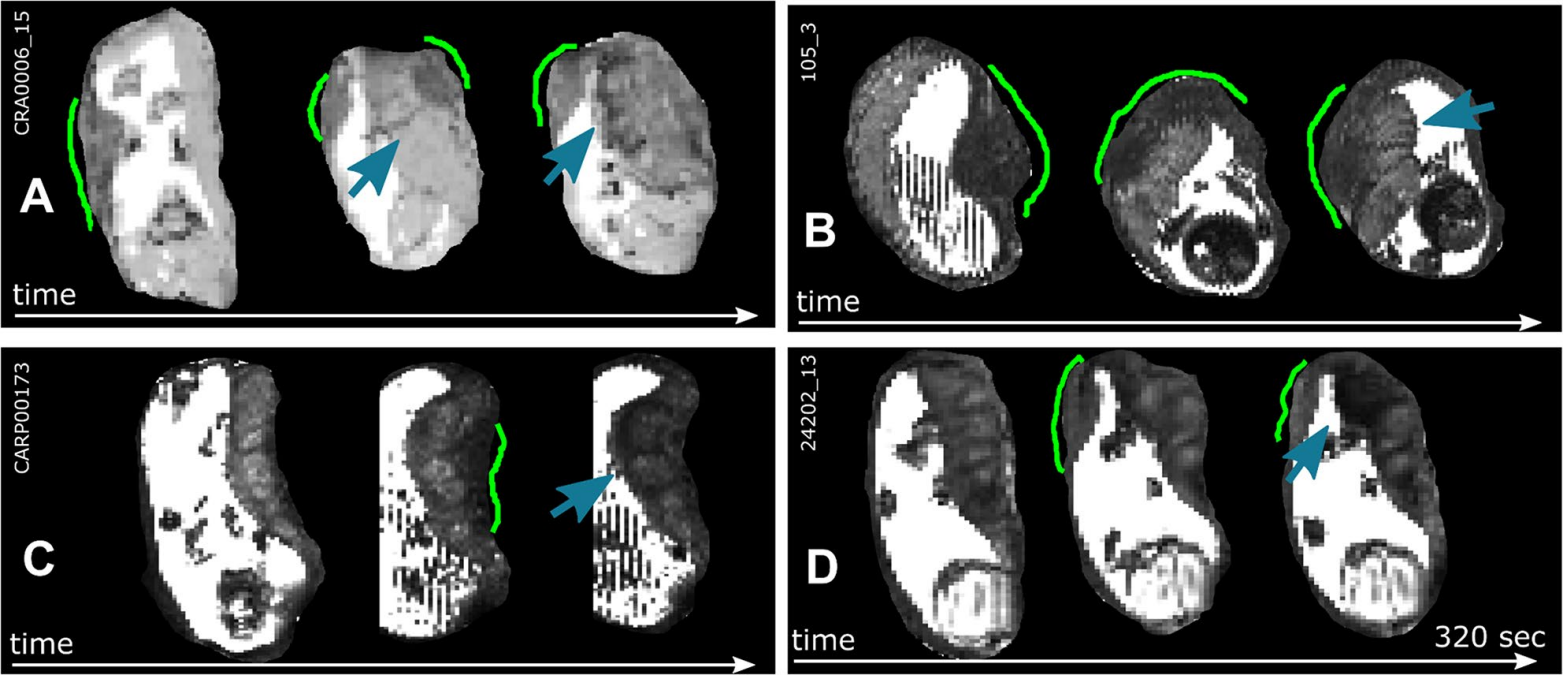
Dynamic time courses illustrating the different characteristics. T2* maps obtained at the same slice at different time points are displayed for subjects 15 (**A**), 2 (**B**), 4 (**C**) and 6 (**D**). Three dynamics over each scan are given in a sagittal direction to the mother, illustrating different phases before and during a uterine contraction. The area of the uterine wall contraction is labelled in green and the visible consequences in the placental T2* map (dark bands and a reduction in signal towards the chorionic plate) are highlighted with blue arrows.

The acquisition of multiple dynamics allows the observation of changes between these states over time, with clear changes observed, for example in Fig. 3A with evolving appearances consistent with criteria for (d), (b–c) and finally (a) within less than three minutes. The thickened area corresponding to the myometrium is marked with green lines and can be observed moving around the uterine wall. Supporting videos S1-S7 show further such contractile events. Both tvisual placental T2* map and the placental volume is initially unchanged while the myometrial thickening is located on a part of the uterine wall not occupied by the placenta. The location of the thickened myometrium moves from the left side upwards to the fundus of the uterus, with dark bands now evident within the placenta and finally a state where no visible myometrial thickening can be observed between placental tissue and uterine wall, but there is now a hypointense band running along the chorionic plate (blue arrows). Similar behaviour and change of these characteristics from (d) to (a) to (b–c) can be seen in Fig. 3B, Fig. 3C displays states (a) and (b–c) during the observation and finally Fig. 3D involves (d) and (b–c). An overview over the assessed criteria can be observed for all selected contractile events in Table 2. Hypointense, thickened areas in either the myometrium underlying the placenta or in other uterine areas can be observed in all but two of the considered cases. The latter, however, both exhibit incomplete coverage of the uterus. Furthermore, longitudinal, vessel-like structures can be observed in the myometrium in most observed contraction cases.

### Quantitative results over gestational age

**Figure 4:**
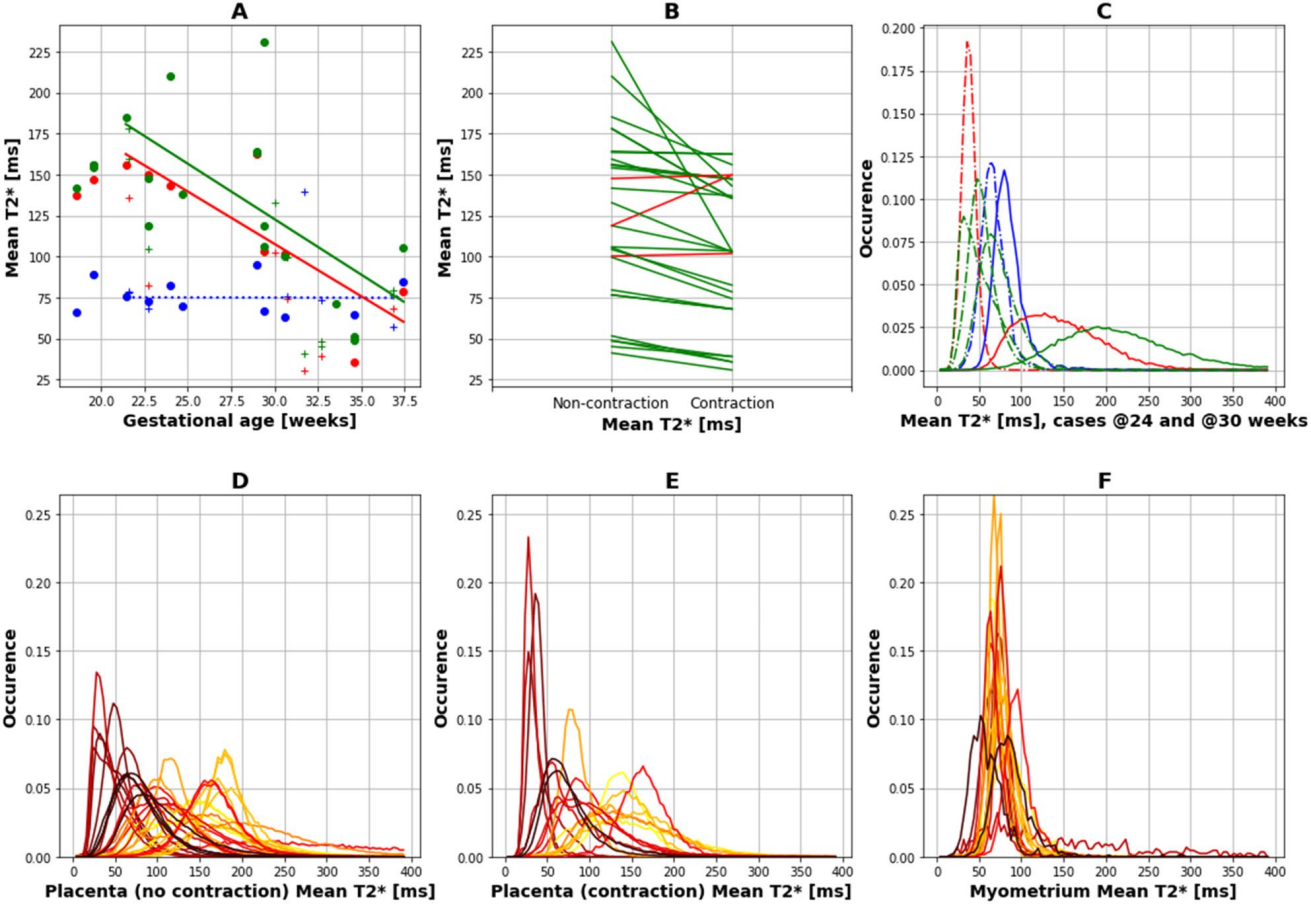
(**A**) Quantitative region-specific results for the detected contractions. Mean results are shown (a) for the placental ROI in a contraction-free time frame (green), in a contractile state (red) and in the underlying myometrium (blue). (**B**) Mean for non-contractile and for contractile placenta regions in green if the non-contractile mean is lower and red if the contractile mean is higher. (**C**) Histograms, depicting the fraction of voxels (occurence) for bins between 1 and 400for two exemplary cases at 24 (continuous line) and 30 weeks (dotted line) are depicted using the same colour coding. (**D**–**F**) Histograms for all subjects are shown for (**D**) non-contractile, (**E**) contractile placenta and (**F**) the myometrium color-coded by gestational age (bright yellow 18 weeks to dark red 37 weeks).

Regional quantitative analysis in the myometrial tissue during the detected contractions and in the placental tissue both during and pre/post contractile activity in the placenta are displayed in Fig. 4. The myometrium outside of contractile activity was too thin to allow stable segmentation, therefore only the myometrium during contractions could be considered. A visual impression of a decrease in T2* over GA both in the contractile and the non-contractile region of the placenta is confirmed by quantitative linear regression of all control results > 20 weeks, the results are given in Table 3. The mean placental T2* in the non-contractile state was significantly negatively correlated to GA (slope = -6.78, @[20,30] weeks = [185,120]ms, *p *= 0.0015). Similarly, the mean T2* values during contractions were significantly negatively correlated to GA (slope = -6.39, @[20,30] weeks = [172,110]ms, *p *= 0.014). The contractile T2* values in the placenta were in all but two cases higher than the measured placental T2* values in the non-contractile state results. The mean T2* results in the myometrial tissue were not related to GA (slope = − 0.02, *p*_value > 0.5) and were between 60 and 100 ms. (B) shows in all but two cases a decline in T2* from non-contractile to contractile state. Histograms illustrating the distribution of T2* values within all three considered areas are shown for two detailed cases in (C), showing the left shift for the contractile curves (in red) as well as the symmetric myometrial distribution centred around 75 ms in both cases (in blue). The curves for all cases are shown for all three areas colour-coded by GA in D-F. The consistent behaviour for all myometrial curves independent of GA is further demonstrated as well as the left shift associated with contractile tissue.

**Table 3:** Overview over the statistical results for the correlation between mean T2* and gestational age [ms].

Region-of-interest	Slope	Values @ 20 and 30 weeks	R-squared and *p*-value
Placenta without contraction	− 6.78 ms/week	[185,120] ms	R-squared: 0.38 *p*-value: 0.015
Placenta during contraction	− 6.39 ms/week	[172,110] ms	R-squared: 0.67 *p*-value: 0.014
Myometrium	− 0.02 ms/week	NA	R-squared: 0.00 *p* value: > 0.5

### Assessing dynamic placental changes

All analysis so far was performed on manually outlined ROIs due to the highly individual and complicated nature of the observed contractions. In the following, dynamic results achieved using automatic processing will be illustrated.

**Figure 5:**
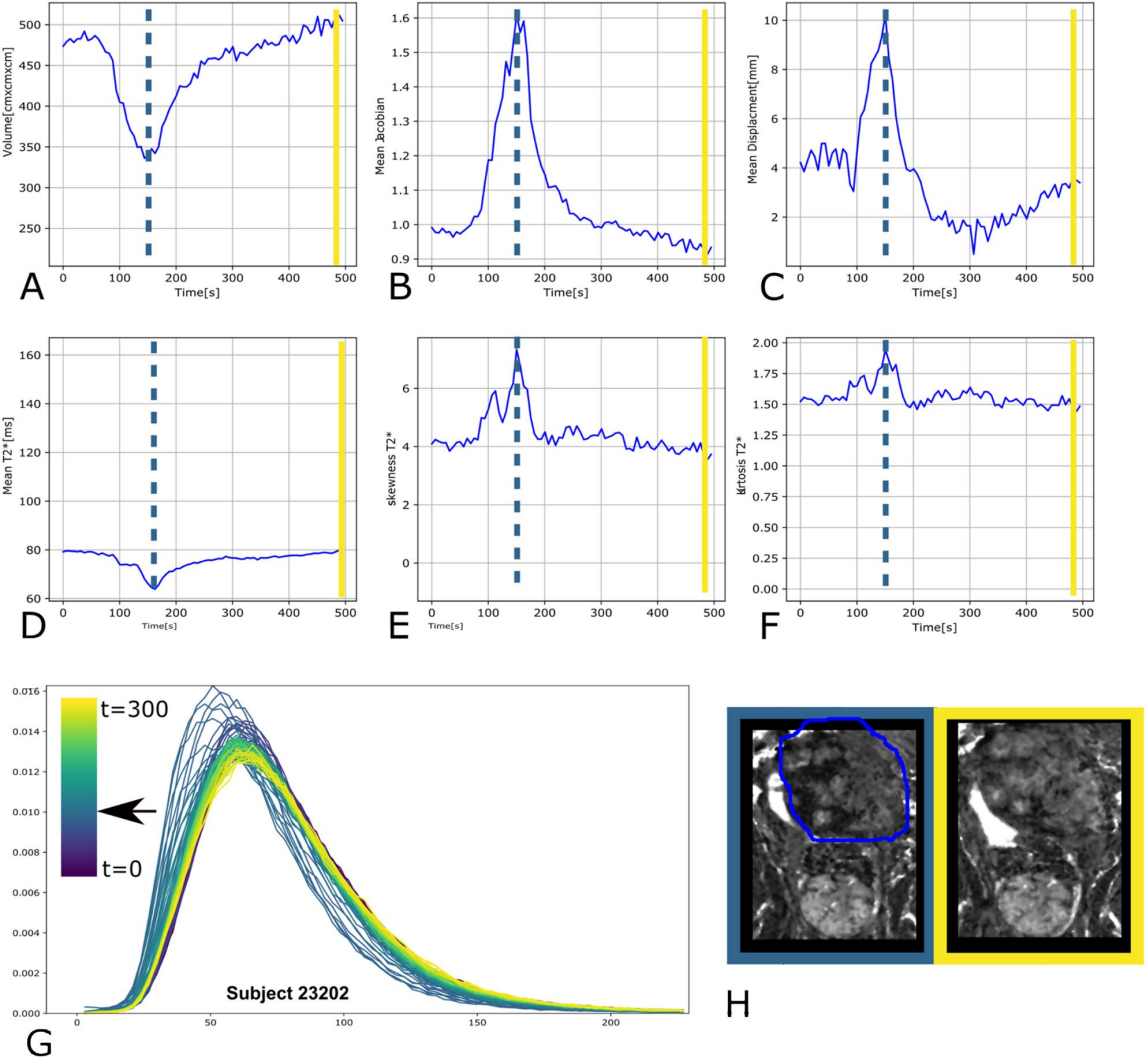
T2* analysis results for subject 1. The time curves for placental T2* are given for the (**A**) volume, (**B**) the mean Jacobian, (**C**) the mean displacement, (**D**) the mean T2*, (**E**) skewness and (**F**) kurtosis. (**G**) The histogram for all dynamics is displayed with the colour indicating the time of acquisition from dark blue to bright yellow and finally (**H**) views during the minimal volume and the maximal volume are given with the ROI indicated for illustrative purposes only. The dashed blue lines indicate the location of the smallest volume, which was assumed to be the most contractile state.

The analysis of the quantitative placental T2* values including the skewness and kurtosis and the mean displacement and Jacobian reveal interesting dynamics. In the example given in Fig. 5, at t = 155 s (dotted line) a simultaneous reduction in placental volume (A) (from 480 to 340 cm^3^, a decrease of 29% ), increase in Jacobian of 60% (B) and in mean displacement of 150% (C), decrease in placental mean T2* from 80 to 64 ms (20% decrease) (D), and increase in skewness and kurtosis in (E,F,) can be observed. All changes reverted slowly until t = 300 s and fit the pattern expected from a contraction.

A similar analysis for cases without contractile activity can be seen in supporting Figure S1 for reference. Similar findings were observed in multiple cases with contractions (see supporting Figures S2 and S3). However, there are cases with contractions without observed changes in volume and multiple datasets where the time course of the contraction—as defined from starting at baseline and returning to baseline—was not complete.

## Discussion

Our study shows both qualitatively and quantitatively the effect of pre-labour uterine contractions on the placenta and uterus. Quantification of the structural deformation of the placenta was attempted but did not adequately identify all contractions. Functional changes in T2* were however demonstrated and measured in both the placenta and the underlying placental bed.

The decrease in T2* values in the placenta with gestational age in the absence of contractile activity (Fig. 5) is in-line with previous studies^6,10^. Similarly, a drop in T2* within the utero-placental unit during contractions, as revealed in previous studies, was confirmed^2,15,17,18^.

A sharp delineation was observed during contractions between the higher signal intensities in the placenta and the hypointensities in underlying, thickened myometrial tissue, with dark, longitudinal structures. This would be consistent with the muscular layer in the myometrium and the arcuate and basal arteriesbranching off from the uterine arteries. Published images available from the third stage of labour^20^ show a similar hypointense tissue with vessel-like hyperintensities remaining in the uterus and the separated placental tissue showing higher intensity. The obtained mean myometrial T2* values in the present study (mean 80 ms) are higher than previously published T2* values in the junctional zone (43.6 + 4.9) and in the peripheral myometrium (63.3 + 5.5)^21^—an increased vascularization in the pregnant uterus and especially in the area underlying the placenta might explain this. Recently shown reduced myometrial T2* during menstruation^22^, would furthermore be consistent.

While the myometrium at first glance appears as a low signal intensity area, hence with reduced T2*, which is in contradiction to other muscular studies showing consistently an increase in T2* during muscular contraction^23^, this observation might reflect the increase in thickness of the myometrium which is too thin in the non-contractile state to be reliably quantified on the T2* maps.

Our data revealed highly dynamic changes, both in terms of the location of the thickened myometrium as well as with regard to the changes in T2* within the placenta. Different characteristics as outlined above were identified (Fig. 2) based on these observations, some similar to patterns suggested in recent work^17^. This study constitutes however the first attempt at quantifying these changes both in the myometrial tissue and placenta. Our dynamic time courses show that the placental appearance changes rapidly and consistently during a contraction reflecting evolution of the underlying tissue composition and oxygenation This might be consistent with the articulated theory of a placental pump activity, which may relate to the recently described uterine pacemaker and/or serve to replenish the oxygen supply to the placental tissue following a contraction.

Research conducted by Oosterhof et al*.* identified increased resistance to flow in the maternal uterine arteries during Braxton Hicks contractions^3^. The spiral endometrial arteries which bathe the chorionic villi are end arteries of the uterine arteries. Thus, increased vascular resistance during subclinical contractions may be reducing uteroplacental perfusion which could explain the hypointense signal intensity observed within the placental tissue. The observed decrease in placental volume might be both due to tissue density changes due to the muscular activity as well as to reduced inflow and increased venous outflow.

The indicated reduced T2*, suggesting as discussed, a decrease in placental oxygenation during the observed contractions, and potentially therefore a decreasing ability to balance maternal supply and feto-placental demand during contractions, as recently highlighted (Burton et al. 2021). The presented insights and the ability to characterise a marker for placental function during contractile activity could enable late gestation assessment and allow insights into the fetal capacity to cope with labour.

Our cohorts seem to suggest an increased occurrence of contractile activity in pregnancies associated with diabetes, this however remains only anecdotal evidence and needs to be verified in larger studies.

### Limitations

The results on contractility and thus the ability to draw wide-ranging conclusions are limited by the number of participants with recorded contractions during the scan. Systematic studies confirming the frequency and effect of subclinical and potentially clinical uterine contractions are thus a necessary next step. Imaging at late gestation may increase numbers with contractile activity and also have more relevance to contractions occurring in labour.

For this study only contractions during the multi-dynamic MEGE scan were analysed and led to the cohort definition. This was chosen for consistency, additional contractions could potentially be found by assessing all the other acquired sequences.

This study used volumetric assessments of the placenta from T2* sequences with echo planar imaging, which may be associated with geometric distortions, hence absolute anatomical volumes are less accurate and can only be interpreted as relative changes over time within one sequence, as seen during a contraction. In addition the whole uterine cavity was not always observed making it difficult to determine whether or not the contractions observed here were often purely underlying the placenta.

Given the intricacy of the uterine-placental interface and the high degree of variation with regard to location, size, thickness, visibility and image quality, a manual segmentation technique was chosen. However, there was strong agreement between the segmented T2* values of the same operator and strong agreement between two independent operators. The close proximity of the placenta to vessels such as the abdominal aorta and its bifurcation, in certain slices, could have modified the local T_2_* value due to partial voluming.

Multiple factors can impact the T_2_* values. Especially the blood flow, which we expect to be altered during contractile activity, and would influence the T2* values. No external recording device, such as a cardiotocograph, was utilised to confirm uterine contractions. Future development of MR-compatible versions of such devices or possible use of e.g. MR-compatible ECG devices will offer additional capacities. Therefore, it cannot be asserted with absolute certainty that all changes observed in the placental tissue were exclusively due to a Braxton-Hicks contraction. This study lacked any measurements of intrauterine pressure which would confirm the amplitude of these contractions. However, the T2* independent measures of shape changes and deformation of the uterus serve as a verification of the visual deformation assessment.

### Implications

The findings and insights of this study could contribute to efforts devoted to reducing the occurrence of late stillbirths and fetal brain injury acquired during birth. Understanding the processes involved in uterine contractions and studying the effects on placental oxygenation might allow the prediction of the coping responses of an individual fetus to both pre-labour and labour contractions. However, it is important to keep in mind that labour contractions are a related, but different phenomenon. The increased strength of labour contractions potentially might increase the trends observed during the Braxton-Hicks contractions—whereby these would indeed carry the potential to predict oxygenation changes in the placenta during labour as discussed above.

In addition to the benefit of this indirect insight into labour, assessing placental function both statically and dynamically during pregnancy allows assessment of its reserve capacity. This may be of particular value in placentas affected by inadequate remodelling of the spiral arteries as seen in pregnancies with pre-eclampsia and fetal growth restriction which may have structural and functional changes as demonstrated in recent MRI studies^7,11^ and which may struggle to maintain the constant supply of oxygen required by the fetus. The effect of contractile activity in such circumstances is thus of high relevance. In addition, recognition of the presence of contractions that may temporarily alter anatomy and function in the placenta is important for any study of the placenta, especially for quantitative measures to ensure consistent reproducible data.

The analysed and aggregated datasets from the current study are available upon reasonable request from the corresponding author.

## Conclusion

This study provides new insights into subclinical contractions in-vivo and thus can pave the way for further studies investigating how this approach may be used for future predictive studies.

The described characteristics and the highly dynamic nature of the changes between these stresses the need for careful evaluation of in-vivo T2* placental data. It furthermore draws attention to the requirement for high temporal resolution if attempting to study placental dynamics. A future direction of research could be to study whether fibrin depositions and calcifications alter the placental effects of contractile activity.

## Supplementary Information

Supplementary Video 1.

Supplementary Video 2.

Supplementary Video 3.

Supplementary Video 4.

Supplementary Video 5.

Supplementary Video 6.

Supplementary Video 7.

Supplementary Information 1.

## Data Availability

The datasets used and analysed during the current study are available from the corresponding author on reasonable request for any interested academic researcher.
