# Supplementary material for: Dynamics of T2* and deformation in the placenta and myometrium during pre-labour contractions
Source: Sci Rep. 2022 Nov 3;12:18542. doi: 10.1038/s41598-022-22008-3 (PMC9633703; doi:10.1038/s41598-022-22008-3)
Supplement: Supplementary file 8 — Supplementary Information 1. [file 41598_2022_22008_MOESM8_ESM.docx]

**Supporting material.**

**Supporting video S1:** Video illustrating the dynamic changes in the anatomical images (top row), T2* maps (bottom row) and graphical representation of T2* changes over time (right).

<https://youtu.be/hqnUePm_ngw> (24202)

**Supporting video S2:** Video illustrating the dynamic changes in the anatomical images (top row), T2* maps (bottom row) and graphical representation of T2* changes over time (right).

<https://youtu.be/-FAeeynkMRM> (105)

**Supporting video S3:** Video illustrating the dynamic changes in the anatomical images (top row), T2* maps (bottom row) and graphical representation of T2* changes over time (right).

<https://youtu.be/CbpM0Sg1KQw> (17)

**Supporting video S4:** Video illustrating the dynamic changes in the anatomical images (top row), T2* maps (bottom row) and graphical representation of T2* changes over time (right).

<https://youtu.be/KPFp025hRaE> (231)

**Supporting video S5:** Video illustrating the dynamic changes in the anatomical images (top row), T2* maps (bottom row) and graphical representation of T2* changes over time (right).

<https://youtu.be/Y6RhqxXZgNg> (289)

**Supporting video S6:** Video illustrating the dynamic changes in the anatomical images (top row), T2* maps (bottom row) and graphical representation of T2* changes over time (right).

<https://youtu.be/OKqTbmZC7W8> (9)

**Supporting video S7:** Video illustrating the dynamic changes in the anatomical images (top row), T2* maps (bottom row) and graphical representation of T2* changes over time (right).

<https://youtu.be/mW5rhbS0z-g> (232)


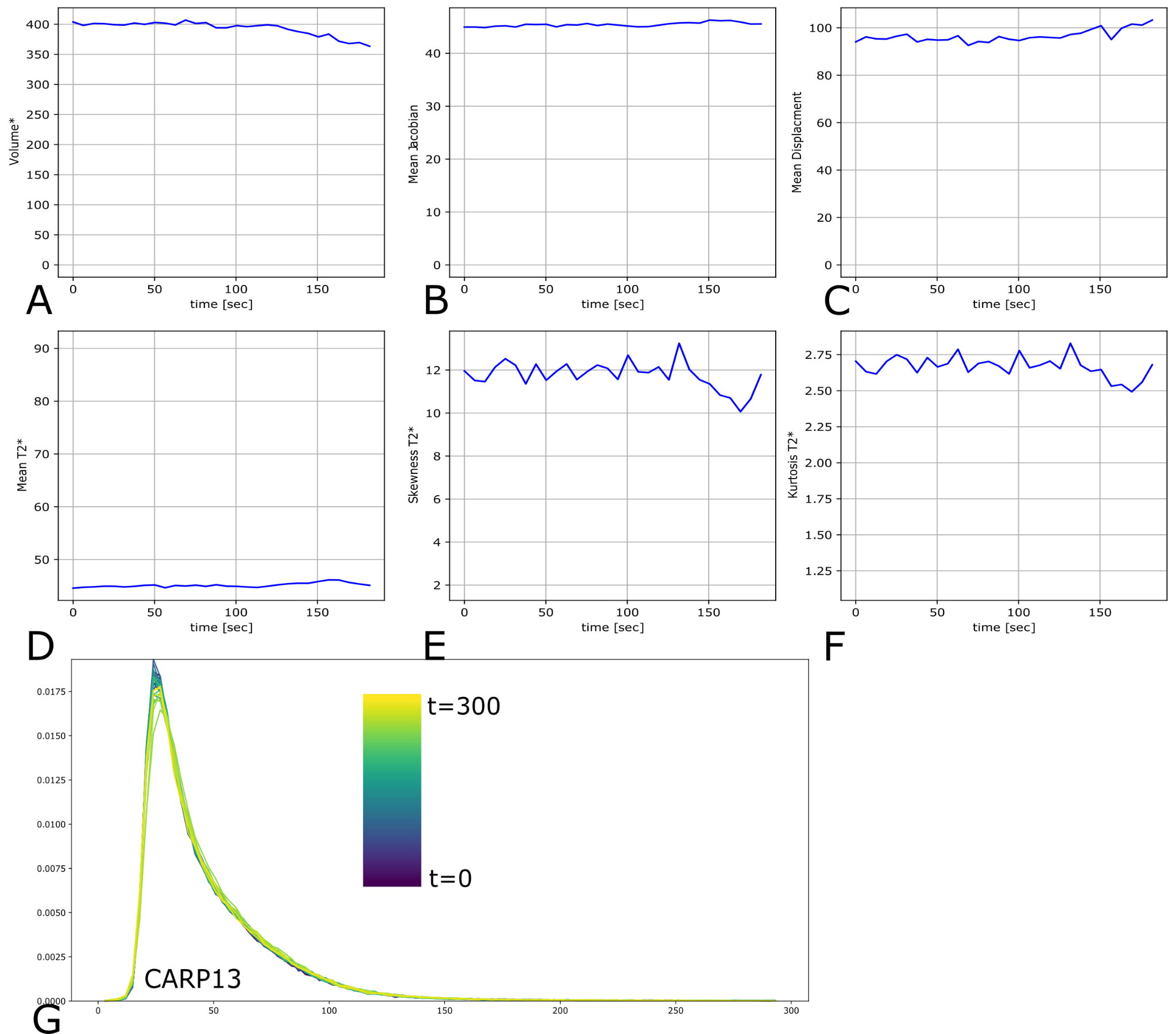


**Supporting Figure S1** Example without contraction. *The time curves for placental T2* are given for the (A) volume, (B) the mean jacobian, (C) the mean displacement, (D) the mean T2*, (E) skewness and (F) kurtosis. (G) The histogram for all dynamics is displayed with the color indicating the time of acquisition from dark blue to bright yellow and finally (H) views during the minimal volume and the maximal volume are given with the ROI indicated.*


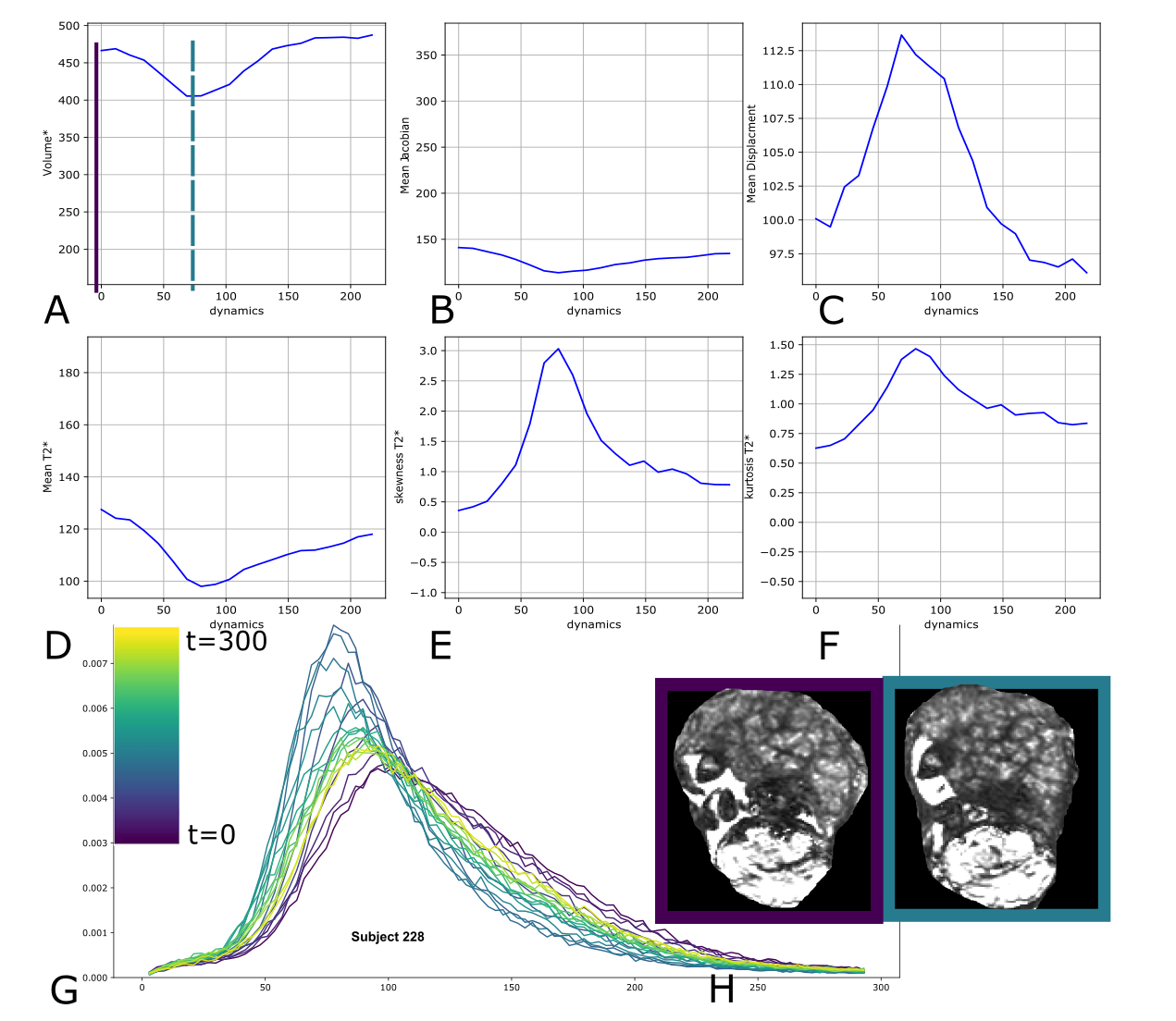


**Supporting Figure S2**- example with a contraction. *The time curves for placental T2* are given for the (A) volume, (B) the mean Jacobian, (C) the mean displacement, (D) the mean T2*, (E) skewness and (F) kurtosis. (G) The histogram for all dynamics is displayed with the color indicating the time of acquisition from dark blue to bright yellow and finally (H) views during the minimal volume and the maximal volume are given with the ROI indicated.*


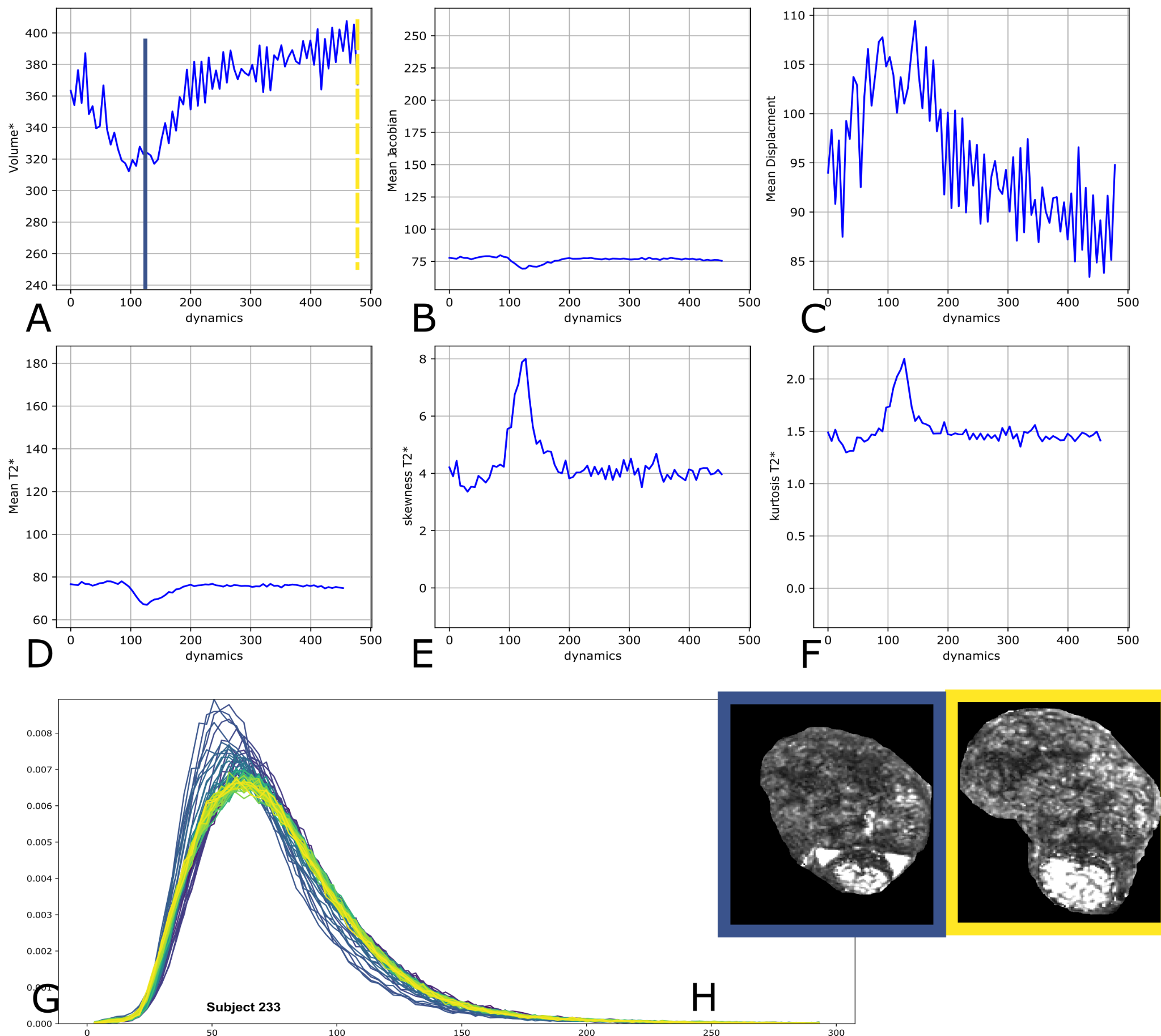


**Supporting Figure S3** - example with a contraction. *The time curves for placental T2* are given for the (A) volume, (B) the mean jacobian, (C) the mean displacement, (D) the mean T2*, (E) skewness and (F) kurtosis. (G) The histogram for all dynamics is displayed with the color indicating the time of acquisition from dark blue to bright yellow and finally (H) views during the minimal volume and the maximal volume are given.*
